# A Novel Synthetic Material, BMM, Accelerates Wound Repair by Stimulating Re-Epithelialization and Fibroblast Activation

**DOI:** 10.3390/ijms19041164

**Published:** 2018-04-11

**Authors:** Ga Young Seo, Changlim Hyun, Dongsoo Koh, Sanggyu Park, Yoongho Lim, Young Mee Kim, Moonjae Cho

**Affiliations:** 1 Department of Biochemistry, School of Medicine, Jeju National University, Jeju 690-756, Korea; rkdud620@jejunu.ac.kr; 2 Department of Pathology, School of Medicine, Jeju National University, Jeju 690-756, Korea; venisua@jejunu.ac.kr; 3 Department of Applied Chemistry, Dongduk Women’s University, Seoul 136-714, Korea; dskoh@dongduk.ac.kr; 4 Division of Life & Environmental Science, Daegu University, Gyeongsangbuk-do 38453, Korea; sgpark@daegu.ac.kr; 5 Division of Bioscience and Biotechnology, Konkuk University, Seoul 05029, Korea; yoongho@konkuk.ac.kr; 6 Institute of Medical Science, Jeju National University, Jeju 690-756, Korea

**Keywords:** wound healing, re-epithelialization, fibroplasia, TGF-β, Cyr61, NADPH oxidase

## Abstract

Cutaneous wound repair is an intricate process whereby the skin reprograms itself after injury. In the mid-phase of wound repair, the proliferation, migration, and differentiation of cells are the major mechanisms to lead remodeling. We investigated the effect of BMM ((1E,2E)-1,2-bis((6-bromo-2H-chromen-3-yl)methylene)hydrazine), a novel synthetic material, on the migration and viability of keratinocytes or fibroblasts using the in vitro scratch woundhealing, electric cell-substrate imedance sensing (ECIS), invasion, and MTT assays. Cell migration-related factors were analyzed using western blot, and we found that treatment with BMM stimulated the EMT pathway and focal adhesion kinase (FAK)/Src signaling. Differentiation of HaCaT keratinocyte and fibroblast cells was also stimulated by BMM and specifically, NOX2/4 contributed to the activation of fibroblasts for wound healing. Furthermore, BMM treated HaCaT keratinocyte and fibroblast-co-cultured cells increased migration and differentiation. TGF-β and Cyr61 were also secreted to a greater extent than in single cultured cells. In vivo experiments showed that treatment with BMM promotes wound closure by promoting re-epithelialization. In this study, we demonstrated that a novel synthetic material, BMM, is capable of promoting wound healing via the stimulation of re-epithelialization in the epidermis and the activation of fibroblasts in the dermis, in particular, via the acceleration of the interaction between the epidermis and dermis.

## 1. Introduction

Wound healing is a complex biological process of restoring damaged skin after injury and is composed of three major phases: inflammation, proliferation, and maturation [1], which are mediated by various cells derived from the epidermis and dermis [2].

In the proliferation phase, basal layer human keratinocytes (HaCaT) move toward the wound site from the edge [3]. HaCaT keratinocytes continue to divide and proliferate to cover the wound area and once complete, the cells cease division and begin to differentiate upward, forming spinous, granular, and cornified layers [4]. This process is known as “re-epithelialization” and is crucial for rapid wound closure. In addition to differentiation, the migration of HaCaT cells also contributes to wound closure through basement layer formation.

Furthermore, dermal fibroblasts (Fbs) crowd around wound sites after injury and form granulation tissue. Myofibroblasts are an activated form of Fbs and are elevated during wound healing [5]. These cells are able to contract by combining smooth muscle actin (SMA) and myosin, and this contraction contributes to wound repair. Extracellular matrix (ECM) components, such as collagens and gelatin, are synthesized from myofibroblasts. Initially, the ECM is composed of collagen-III, which is a weaker form of collagen that is later replaced by the longer formed-collagen-I, as evidenced in scarring [6]. In the late phase of wound repair, the myofibroblasts are lost via the apoptosis pathway and expressed collagen-I is degraded by matrix metalloproteinases (MMPs) to balance the composition of the ECM [7], which is known as the cause of several fibrotic diseases such as cirrhosis, organ fibrosis, or hypertrophic scarring of the skin [8].

Previously, reactive oxygen species (ROS) have been examined for their involvement in dermal fibroblast activation. Sampson et al. demonstrated that dysregulated redox homeostasis underlies myofibroblast differentiation in the prostatic stroma via NOX4-derived signaling [9]. NADPH oxidase is one of the ROS generating enzymes and has six types of subunits.

Several studies have reported a relationship between the epithelial–mesenchymal transition (EMT) and embryogenesis, wound healing, or metastasis. Epithelial cell adhesion molecules (ECAM) function to interlink epithelial cells and usually maintain a fixed form. However, when ECAM synthesis was blocked by Twist, Slug, or Snail transcription factors, cells lost their adhesive ability and transformed into spindle and motile mesenchymal cells types. These mechanisms are fundamentally relevant to the acquisition of cells’ migratory ability and are also related to wound healing [10].

Focal adhesion kinase (FAK) and C-Src form a dual kinase complex and phosphorylate down-stream factors such as the paxillin and p130^Cas^. Integrin-regulated activation of FAK and Src functions to increase cell migration, cycle, and survival in normal cells or leads to tumor growth and metastasis in tumor cells [11].

In this study, we show that BMM, a novel synthetic material, has wound repair effects both in vitro and in vivo. BMM caused HaCaT keratinocytes to have an EMT-like phenotype and migratory effects via the FAK/Src pathway. Dermal fibroblasts were also activated by BMM through the generation of NOX2/4-ROS, and specific cytokines TGF-β or Cyr61 were related to these mechanisms. Specifically, we used a co-culture system that was recently developed in our previous studies to investigate whether BMM stimulates interaction between keratinocytes and dermal fibroblasts [12]. Our results suggest that BMM accelerates cutaneous wound repair by stimulating re-epithelialization and fibroblast activation.

## 2. Results

### 2.1. BMM Increases Migration of HaCaT Cells but Has No Effect on Fbs

**Figure 1:**
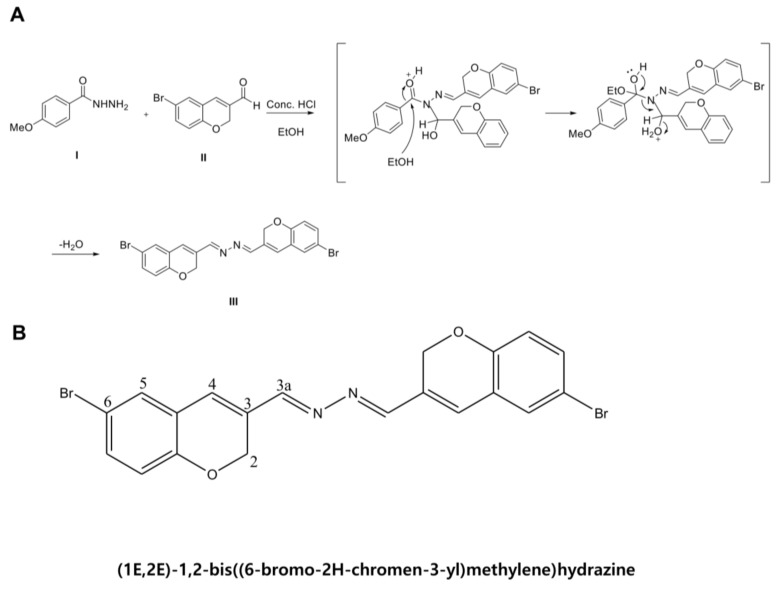
Scheme and chemical structure of BMM. (**A**) Scheme of BMM synthesis; (**B**) The structure and numbering of BMM, (1E,2E)-1,2-bis((6-bromo-2H-chromen-3-yl)methylene)hydrazine.

BMM is a DK223 derivate and is an abbreviation of 1E,2E-1,2-bis(6-bromo-2H-chromen-3-yl)methylene]hydrazine (Figure 1). In a previous study, Manh Tin Ho et al. demonstrated that novel compound DK223 has wound healing effects via ROS production in HaCaTs and TGF-β induction in Fbs [13]. We synthesized several derivatives of DK223, compared the wound healing activities, and selected BMM, which is easier to produce and has a low cost unit price.

**Figure 2:**
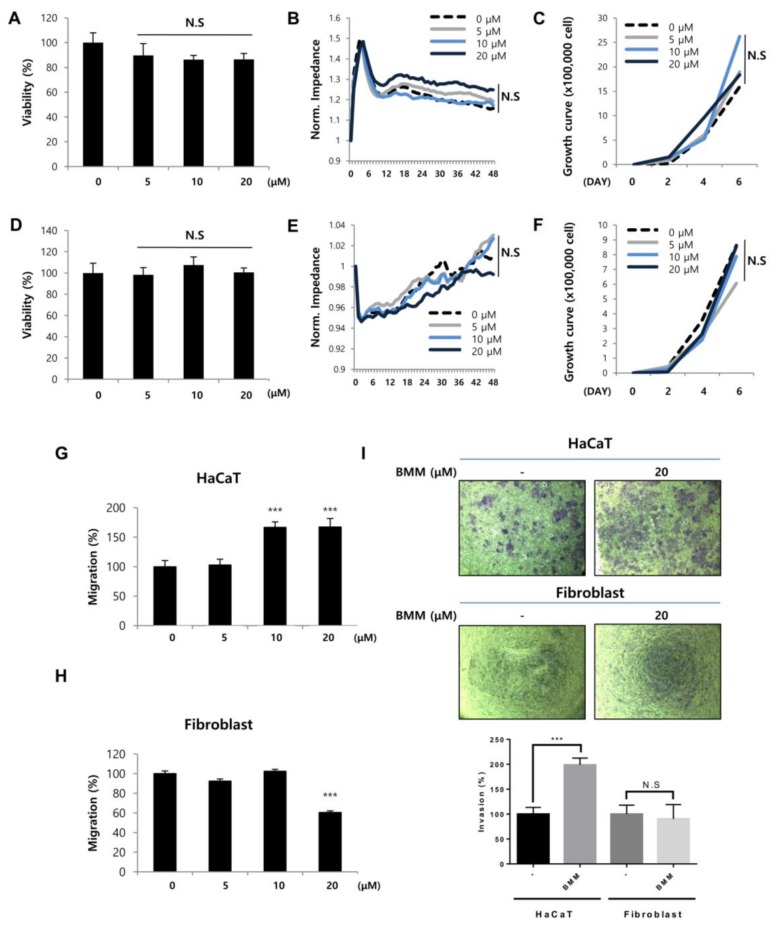
BMM increases basal layer human keratinocytes (HaCaTs) migration but not proliferation and has no effect on proliferation and migration of dermal fibroblasts (Fbs). (**A**) Viability of HaCaTs and (**D**) Fbs. Cells (2 × 10^4^ cell/mL) were seeded and treated with BMM (0–20 μM). After incubation for 24 and 48 h, viability of cells was measured using the MTT solution (5 mg/mL). N.S, not significant; (**B**) Detection of real time proliferation of HaCaTs and (**E**) Fbs treated with BMM. Cells (2 × 10^4^ cell/mL) were seeded into eight-well plates pre-coated with 10 mM l-cysteine (Electrode-stabilizing solution). Cells were treated with 5–20 μM of BMM and measurement was started at 48 h by using the ECIS machine. Resistance was calculated with a ratio compared to 0 h. N.S, not significant; (**C**) Cell counting assay of HaCaT and (**F**) Fbs treated with BMM (0–20 μM). Cells were seeded (5 × 10^3^ cells/well) of on six-well plates and the following day, cells were treated with various concentrations of BMM. After two, four, and six days, cells were collected using 1 × Trypsin and counted using a hemocytometer after centrifugation. N.S, not significant; (**G**) Scratch wound healing assay with HaCaT and (**H**) Fbs treated as described in (**A**) 48 h post-treatment. Migration distance was measured using the ImageJ program; (**I**) Transwell invasion assay with HaCaTs and Fbs treated with 20 μM BMM for 48 h; Invasive ability was measured as described in the Materials and Methods and is quantified under the graph. *** *p* < 0.001 as compared to that of the control, N.S, not significant.

To investigate the effect of BMM on HaCaTs or Fbs, we examined migratory and proliferative effects using scratch wound healing and proliferation-related assays, respectively. Migration of HaCaTs and proliferation of Fbs are key biological processes during various steps of wound healing, including re-epithelialization and matrix composition. The effects of BMM on the proliferation of HaCaT and Fb cells, which were analyzed by MTT (Figure 2A,D), ECIS (Figure 2B,E), and cell counting (Figure 2C,F) assays, did not show any significant dose-dependent change, and accordingly, it seems that BMM has no toxicity on HaCaTs and Fbs.

However, migration, which is a measurement of distance of mobile cells, and invasion, which is measurement for penetrating ability by forming lamellipodia, of HaCaT cells treated with BMM, were maximally increased after treatment with 20 μM BMM (Figure 2G,I) and thus we chose this as the optimum concentration for use in subsequent experiments. The effect of BMM on Fbs seemed to be lacking (Figure 2H,I). These results indicate that BMM effects the migration of HaCaTs but not Fbs.

### 2.2. HaCaTs Have an EMT-Like Characteristic and Promote MMP Secretion by BMM Treatment

**Figure 3:**
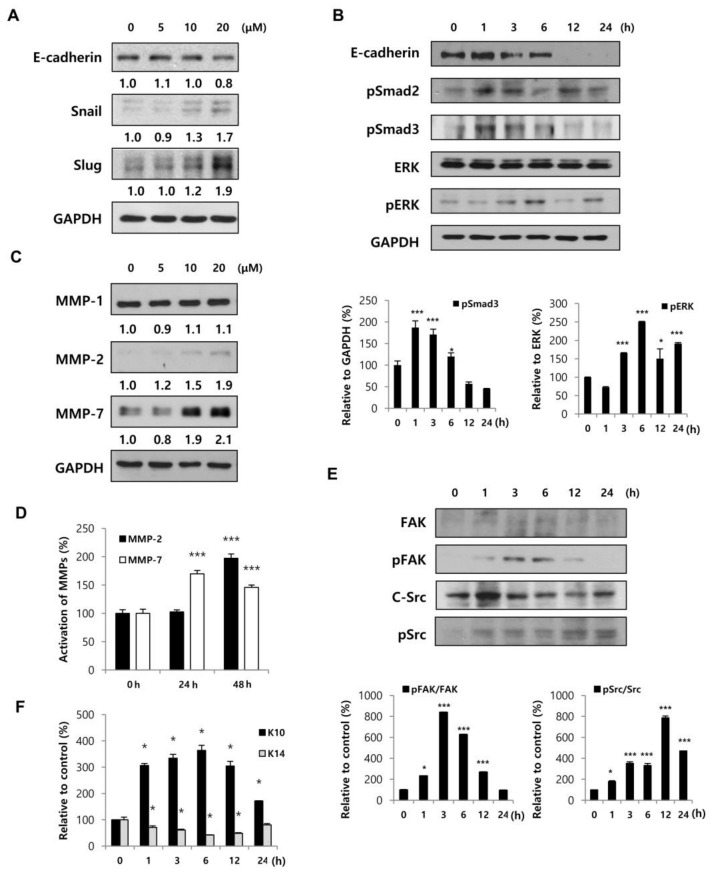
BMM promotes HaCaT cell migration by inducing an EMT-like phenotype and FAK/Src pathway and stimulates differentiation. (**A**) HaCaT cells (1 × 10^5^ each) were seeded on 100 mm cell culture dishes and incubated for 24 h; Protein lysates from cells treated with BMM in a dose-dependent and (**B**) time-dependent manner were collected using RIPA solution, and proteins were resolved by 10% sodium dodecyl sulfate-polyacrylamide gel electrophoresis (SDS-PAGE). Western blot to detect the expression of EMT-related factors was performed, and expression levels were normalized to GAPDH. The values indicate intensities of protein expression with respect to that of the loading control; (**C**) Western blotting for HaCaT cells treated with BMM for 24 h and MMP expression was measured. The expression levels were normalized to GAPDH. The values indicate intensities of protein expression with respect to that of the loading control; (**D**) Western blotting for secretion of MMPs from BMM-treated-HaCaT cells. Conditioned medium was concentrated using an Amicon centrifugal filter and total protein concentration was measured by the Bradford assay. The graph indicates expression levels normalized to PonceauS, as a loading control; (**E**) Western blot to measure the expression of phospho-FAK and phospho-Src as a mechanism of migration in BMM-treated HaCaT cells. The activation of the FAK/Src pathway was calculated by comparing with total-FAK and total-Src; (**F**) Western blot assay to detect differentiation and proliferation of HaCaT cells, which are respectively marked with K10 and K14, after BMM treatment, and in all cases, expression levels were normalized to GAPDH. * *p* < 0.05 and *** *p* < 0.001 as compared to the control.

Cell adherent molecules (CAM), such as E-cadherin, function to form tight junctions between cells and Snail, Twist, or Slug, which are transcription factors, blocking the synthesis of these molecules. As a result of changes of these molecules, the epithelial cells are transformed into a mesenchymal type and gain migratory ability; this is known as the EMT. Thus, we attempted to analyze whether the EMT related to HaCaT migration is affected by BMM. When cells were treated with BMM in a dose-dependent manner, E-cadherin expression at 20 μM was down-regulated by 80% compared to that of the control, and levels of both Snail and Slug were significantly increased, especially at 20 μM (Figure 3A). To confirm the mechanism of EMT action by BMM, we treated HaCaT cells time-dependently. Smad2/3 is known to be phosphorylated as a result of TGF-β stimulation through a canonical pathway and ERK phosphorylation is constrained to the TGF-β-stimulated non-canonical pathway. Phosphorylation of Smad2/3 and ERK peaked until 1 and 6 h after treatment and then decreased (Figure 3B). Down-regulated E-cadherin latterly might be affected by upstream stimulation, in both the canonical and non-canonical pathways.

Along with the EMT process, MMPs also contribute to cell migration [14]. It is known that MMPs degrade ECM components such as collagen and gelatin. The expression of MMP-2 and MMP-7, but not MMP-1, was dependently increased (Figure 3C) and the detection of their secretion in conditioned medium was also doubly increased (Figure 3D). These results indicate that BMM promotes HaCaT migration through EMT stimulation and MMP secretion.

### 2.3. BMM Induced HaCaT Migration through FAK/Src Activation and Promotes Cell Differentiation

Various signaling pathways related to cell migration have been reported, including that of the FAK/Src pathway. FAK stimulated by several stimuli forms a complex with Src and activates the down-stream pathway by phosphorylation, for the regulation of cellular functions, especially migration and invasion [15]. As shown in Figure 3E, phosphorylation of FAK peaked at 6 h after BMM treatment and then decreased time-dependently. Phospho-Src was also increased and sustained until 24 h after treatment. These results showed that BMM promotes the migratory effect of HaCaTs through the FAK/Src pathway.

Next, we analyzed whether BMM affects the proliferation or differentiation of HaCaT cells. Keratin 10 is specifically expressed in the epidermal layer except the stratum basal and can be used as a marker of differentiated epidermal cells [16]. In Figure 3F, BMM promotes differentiation of HaCaTs and surprisingly, keratin 14 expression, which is a marker of proliferation in the basal layer, was conflictingly decreased. This is because a fully proliferated basal layer begins to differentiate for stratification upwards. From these experiments, we demonstrated that BMM induces the differentiation of HaCaTs by ending basal layer proliferation and initiating stratification.

### 2.4. Fbs Treated with BMM Are Activated by the NOX4-ROS-CCN1 Pathway

**Figure 4:**
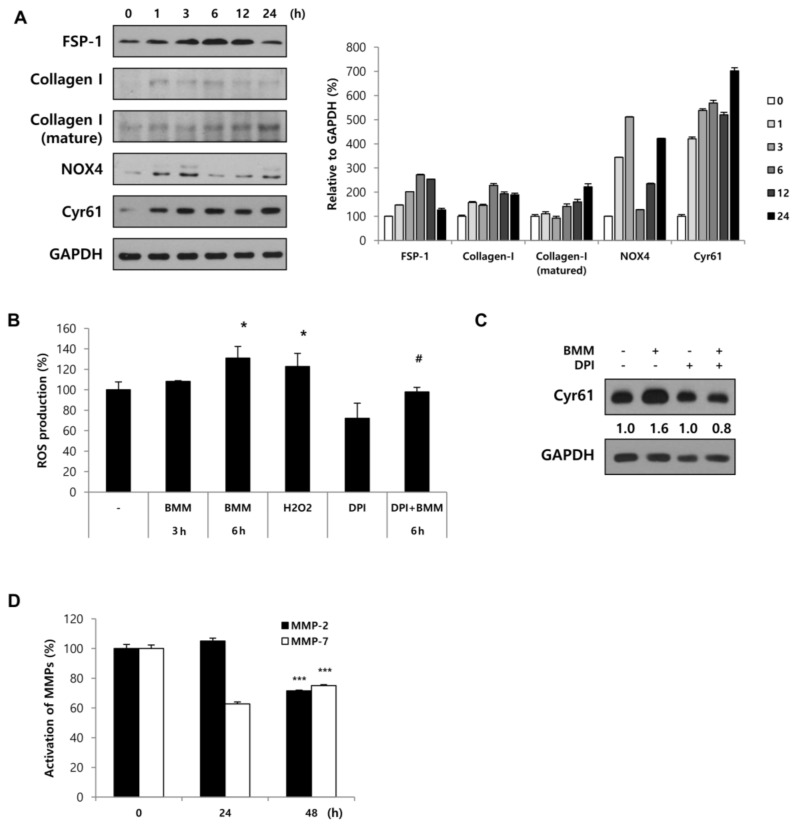
Fbs stimulated by BMM differentiate into myofibroblasts via the Cyr61/Nox4 pathway. (**A**) Western blotting for Fb differentiation, involving analysis of specific factors such as FSP-1, NOX2, NOX4, collagen-1, and Cyr61. The expression of these factors was normalized to that of GAPDH; (**B**) Cells were treated with BMM for 3 or 6 h or DPI (5 μM; NOX inhibitor) for 1 h or were co-treated with DPI and BMM. ROS production was measured using the DCF-DA assay and calculated as a percentage of the mean fluorescence intensity compared with that of the control. * *p* < 0.05 as compared to the control; # *p* < 0.05 as compared to the BMM (6 h)-treated group; (**C**) Cells were treated with BMM for 3 h or DPI (5 μM, NOX inhibitor) for 1 h or were co-treated with DPI and BMM. Cyr61 expression was normalized to that of GAPDH after western blotting. The values indicate intensities of protein expression with respect to that of the loading control; (**D**) Secretion of MMPs from BMM-treated-Fbs by using conditioned medium, followed by western blotting analysis. The expression levels were normalized to PonceauS, used as a loading control. *** *p* < 0.001 as compared to the control.

We have shown that BMM promoted HaCaT migration through the EMT, FAK/Src pathway, and differentiation. In order to determine the effect of BMM on Fbs, we analyzed NOX expression. It has been reported that NADPH oxidases (NOX), which are enzymes that generate ROS, are related to wound healing by activating Fbs [17]. When we treat Fbs with BMM in a time dependent manner, the expression of NOX4 peaks at 3 h and then decreases, whereas NOX2 does not seem to be involved. Cyr61 expression also increased until 3 h and then remained stable until 6 h. Fb activation was induced slightly at 3 h and collagen synthesis was also increased following dermal activation (Figure 4A). This indicates that BMM activates Fbs mainly through NOX4 and modulates collagen synthesis. In addition to collagen synthesis, it has been shown that ROS produced by NADPH oxidases upregulate CCN1 expression. Qin et al. found that CCN1 expression is not only promoted by increased intracellular levels of ROS, but is also induced by oxidative stress, and this positive feedback loop continues to generate ROS and increase CCN1 in Fbs [18]. As shown in Figure 4A, NOX4 expression peaked at 3 h after BMM treatment, resulting in elevated Cyr61 expression at 3–6 h. This finding indicates that ROS generation due to NOX4 mediated Cyr61 expression. Next, we investigated whether the increase in ROS due to NOX4 directly affects Cyr61 expression at early time points. For this, Fbs were treated with BMM for 3 h, which was the optimal time point for NOX4 expression, or with diphenyleneiodonium (DPI), a NOX inhibitor. The 2′,7′-dichlorofluorescin diacetate (DCF-DA) assay showed that BMM-treated Fbs started generating ROS and that the production peaked at 6 h. ROS production was reduced by the NOX inhibitor DPI, which indicated that Fbs treated with BMM produce ROS in a NOX4-dependent manner (Figure 4B); the increase in Cyr61 expression at 3 h due to BMM treatment was also blocked by the NOX4 inhibitor (Figure 4C). These results indicate that there is an indirect relationship between NOX4 and Cyr61 in the early stages (i.e., at 3 h after treatment), and we speculate that ROS regenerated by NOX4 promotes Cyr61 expression through secondary ROS signaling, thereby forming a positive feedback loop with Cyr61-ROS.

In addition to dermal activation by NOX-ROS-CCN1, we analyzed the secretion of MMP-2 and MMP-7, which are known to degrade ECM components of Fbs. Expression of matured MMP-2 and MMP-7 in the media were decreased as a result of BMM treatment (Figure 4C). From these experiments, we demonstrated that BMM stimulates the differentiation of Fbs through Cyr61 and NOX4 activation and contributes to the synthesis of collagen-I for dermal contraction. It also inhibited the secretion of MMP-2/7.

### 2.5. BMM Promotes Migration to a Greater Extend in Co-Culture Than in Single Culture

**Figure 5:**
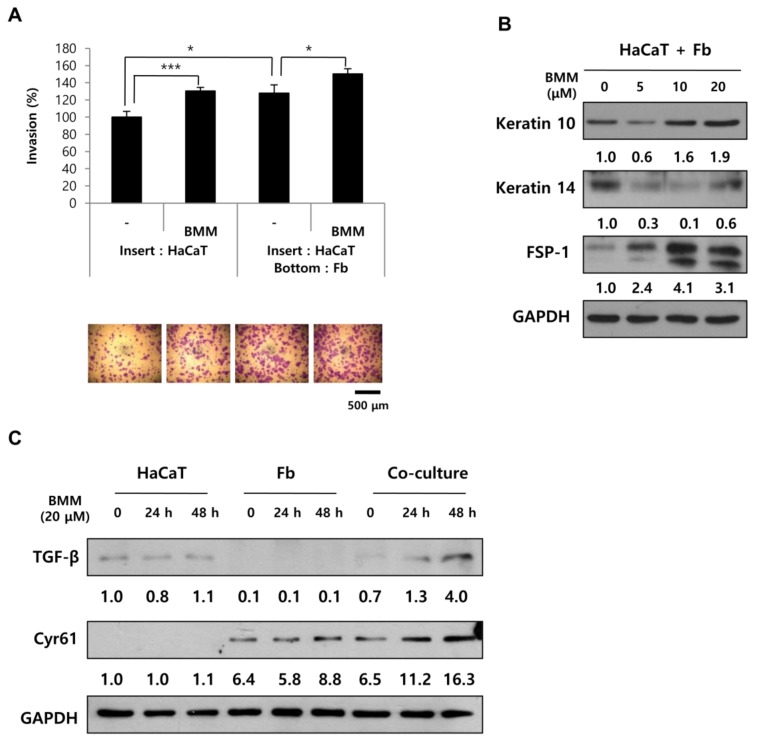
In co-culture, BMM further stimulates the migratory ability of HaCaT cells through the secretion of TGF-β and Cyr61 from HaCaT and Fbs, respectively. (**A**) Transwell invasion assay of HaCaT cells after BMM treatment. For single culture, only 7 × 10^4^ cells of HaCaT were seeded on the insert, and an equal number of Fbs were cultured on the bottom at the same time for co-culture. After crystal violet staining, invasive ability was measured using the ImageJ program; (**B**) Western blotting for co-cultured cells to detect differentiation of HaCaT (K10) or Fbs cells (FSP-1). The values indicate intensities of protein expression with respect to that of the loading control; (**C**) The expression of TGF-β and Cyr61 on HaCaT, Fbs, and co-cultured cells using western blotting. The values indicate intensities of protein expression with respect to that of the loading control. * *p* < 0.05 and *** *p* < 0.001 as compared to the control.

Since the data indicates that BMM has effects on HaCaT and Fb cells, we wanted to confirm whether the interaction between HaCaT and Fbs is affected by BMM. A transwell plate, having an 8 μm pore polycarbonate membrane, was used for co-culture to enable the sharing of media between HaCaTs and Fbs [12]. By measuring the amount of cells migrating to the lower chamber, we can determine the migratory ability and specifically, the invasion effect, which is the penetrating capability through a pore membrane by forming cell protrusions. We assessed the migratory effect using a transwell-invasion assay kit and found that HaCaTs co-cultured with Fbs showed faster movement than HaCaTs cultured alone. Interestingly, BMM promotes the migration of both HaCaT-single and co-cultured cells to a greater extent than those treated with the control (Figure 5A). This indicates that BMM regulates the interaction between these cells, as well as that of HaCaT or Fbs alone. We assumed that the major regulatory factors are TGF-β and Cyr61. Based on Figure 4A, Cyr61 is expressed in Fbs and its expression is stimulated by BMM. The expression of Cyr61 was not detected in HaCaT cells despite BMM treatment, but was significantly increased in Fb and co-culture conditions in a time-dependent manner. TGF-β has also been reported to be involved in metastasis, cell migration, ROS production, and wound healing [19]. The expression of TGF-β was slightly increased by BMM only in HaCaT cells and surprisingly, it was further increased in co-cultured cells similar to that observed for Cyr61 (Figure 5B). These data indicate that HaCaTs and Fbs are stimulated under co-culture conditions, which results in elevated cell migration and demonstrates, in particular, that TGF-β secreted from HaCaTs and Cyr61 secreted from Fbs may affect mutual cells for this synergetic effect (Figure 5B). Epidermal differentiation, indicated by upregulation of keratin 10, was also induced in co-cultured cells along with proliferation, labeled with keratin 14, inhibition, as well as dermal activation (FSP-1) (Figure 5C). 

### 2.6. BMM Accelerates Wound Healing

**Figure 6:**
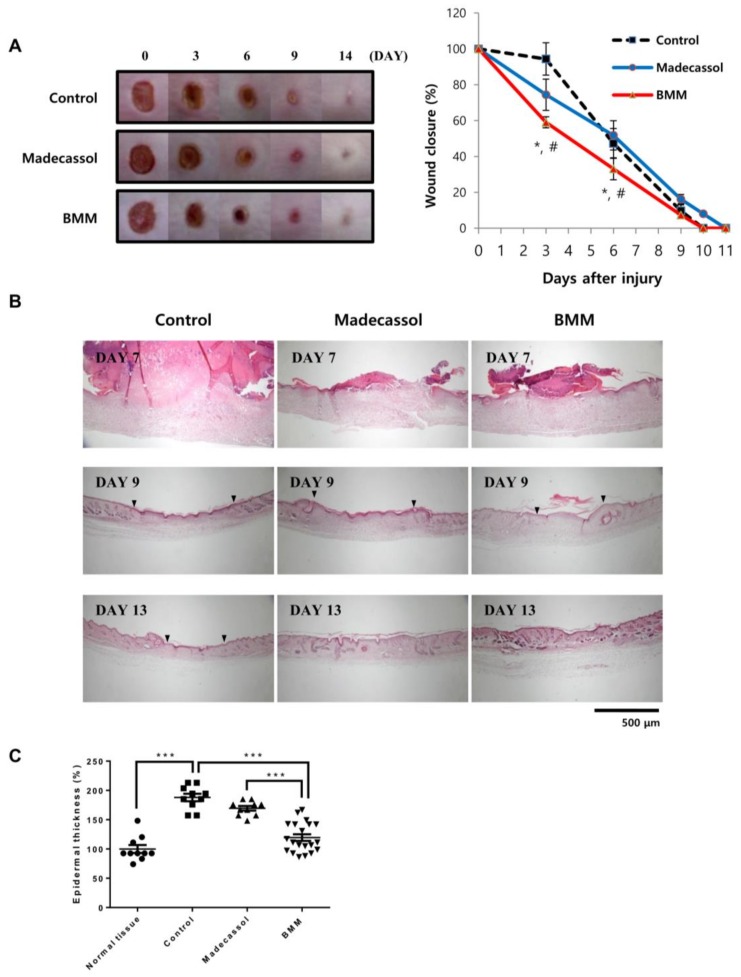
BMM accelerates wound healing in an in vivo excisional wound model. (**A**) Wound closure in an excisional wound model over 14 days. Photographs were taken every three days, and wound size was measured using the ImageJ software. ‘Control’ indicates the butylene glycol and “Madecassol” was used for a positive control. The rate of wound closure is shown as a graph. * *p* < 0.05 as compared to the control, and # *p* < 0.05 as compared to Madecassol; (**B**) Hematoxylin&Eosin staining of skin tissue isolated from the wound site on indicated days; (**C**) Epidermal thickness was measured 13 days after wounding using the Image J program. *** *p* < 0.001. Dotted line; re-epithelialization, arrowhead; wound edge, and arrow; regenerated skin appendages. Epidermal thickness shown as a graph.

Until now, we have shown the effects of BMM on wound repair using skin cells, especially in a co-culture system. We applied this effect to the excisional wound model of mice. Wounds (6 mm of diameter, *n* = 6 for each group) were made on the back of mice and treated with BMM every day. Madecassol, a known healing ointment, was used as a positive control. As shown in Figure 6A, wounds of mice in the BMM treated group healed significantly faster than those of the control and Madecassol groups. Skin tissue stained with Hematoxylin/Eosin indicated fast re-epithelialization in the BMM group, as indicated by the dotted line and the epidermal thickness of the wound site of the BMM group was less than that of the non-wounded skin. The width of the wound (arrowhead) of the BMM group at day nine post-wounding was smaller than that of the other groups and was completely closed at day 13. Newly differentiated appendages (arrow) were also located at the scar site of the BMM group. These data are consistent with previous in vitro results, demonstrating that a novel synthetic material, BMM, possesses a remarkable wound healing ability.

**Figure 7:**
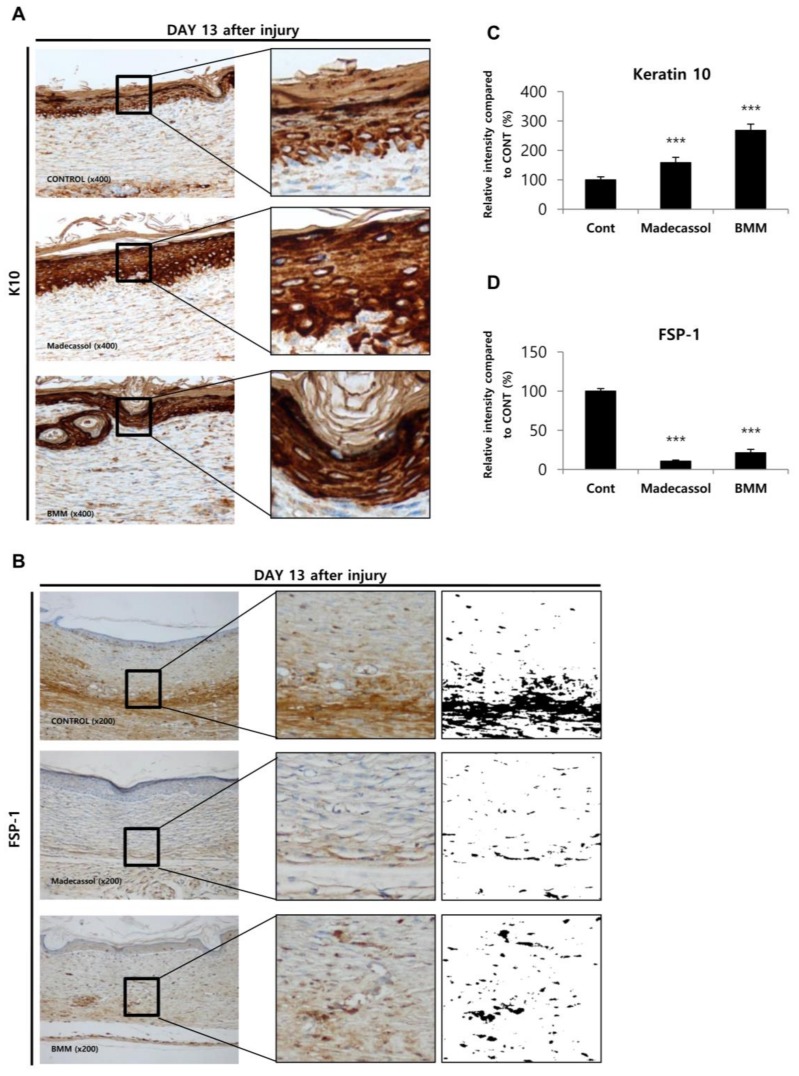
The epithelial restoration and anti-scarring effect of BMM in the late phase of wound healing. (**A**,**B**) Immunohistochemical staining of skin tissue isolated from the wound site on indicated days; (**C**,**D**) Intensities of K10-positive epithelial cells and FSP1-positive fibroblast cells were measured using the ImageJ software. “Control” indicates butylene glycol and “Madecassol” was used as a positive control. *** *p* < 0.001 as compared to the control.

Next, we examined whether our in vitro findings correspond to that in vivo using IHC staining of keratin 10 and FSP-1 (Figure 7). As mentioned in Figure 3, keratin 10, a differentiation marker of epithelial cells, shows increased expression upon BMM treatment of HaCaT cells. In in vivo IHC staining, the intensity of K10-positive epithelial cells was significantly increased by BMM (Figure 7A,C), whereas that of FSP-positive fibroblasts, corresponding to the activation of fibroblasts, was decreased by BMM treatment (Figure 7B,D). In the in vitro assay, an increase of FSP-1 expression implied improvement of dermal contraction by BMM via dermal differentiation. However, activated fibroblasts in the wound healing process are reduced to a normal state to reduce fibrosis, resulting in scar formation and balanced collagen synthesis [5]. Through these additional experiments, we further demonstrated that BMM restored the epithelial barrier, as suggested by K10 up-regulation, and has an anti-scarring effect, as indicated by FSP1 down-regulation at a late phase of wound healing, as well as a fast wound closure.

## 3. Discussion

Our study presents evidence that BMM treatment can accelerate cutaneous wound healing both in vitro and in vivo. BMM is a novel synthetic compound derived from DK223, which has been previously proven as a potential therapeutic agent for wound repair [13]. BMM is derived from DK223 by the substitution of a methoxy group to a bromo group. Unlike DK223, BMM only has an effect on HaCaT migration, not proliferation, and Fb proliferation (Figure 2).

The epidermal layer functions as a barrier of the outside skin and consists of several layers, such as cornified, granular, spinous, and basal layers that are respectively marked with loricrin, keratin 1/2/10, transglutaminase 1/5, and keratin 5/14/15 [20]. It is the “re-epithelialization” that covers damaged skin with newly divided keratinocytes through migration along the dermis. After the cells meet beneath the scab, the scab is disconnected from the skin and epithelial cells continue to differentiate, approaching the normal thickness of the epidermis [21]. We have found that this phenomenon occurred in HaCaT cells treated with BMM at an optimum concentration of 20 μM. While BMM has no effect on HaCaT proliferation, migratory ability was increased maximally two-fold. Our results showed that the underlying molecular mechanism to promote HaCaT cell migration results from multiple processes including the EMT, FAK/Src pathway, and MMP secretion. Samy Lamouille et al. have reported that the EMT, a process initiated by TGF-β, is related to wound healing, as well as development, fibrosis, and cancer progression by inducing cell motility [22]. The epithelial marker, E-cadherin, was significantly decreased and the mesenchymal markers Snail and Slug were dose-dependently increased in HaCaT cells treated with BMM. The relationship between the FAK-Src pathway and wound healing was also identified by Zhao et al. [15]. They explain that an active FAKSrc complex regulates membrane ruffling and cell migration via phosphorylation of downstream molecules, such as p130^Cas^, cdc42, or PI3K, and also plays a critical role in angiogenesis [15]. It is known that FAK, which is a focal adhesion-related protein kinase, is mediated by integrins. Autophosphorylation of FAK at tyrosine297 occurs by integrin activation and creates a binding site for the SH2 (Src-homology2) domain of Src, which in turn phosphorylates other tyrosine sites in FAK. The active FAK-Src complex promotes Rac1 and finally stimulates lamellipodia formation. As RhoA is reversibly suppressed by an active FAK-Src complex, the formation of stress fibers decreased. Integrin-mediated FAK-Src activation increases membrane protrusion and decreases contractility, thereby regulating cell migration. We confirmed that BMM stimulates the phosphorylation of FAK at Y397 and Src at Y416 (Figure 4) and speculate that it is a key mechanism for cell mobility. The effect of BMM on MMP expression and secretion was different depending on subunits. The expression of MMP-1, well known as collagenase, was unchanged in spite of BMM treatment. However, the expression of MMP-2 and MMP-7, a gelatinase-A and matrilysin-1, was elevated and further secreted into the medium by BMM. It is known that MMP-2 digests collagen-I, II, and III and MMP-7 hydrolyzes cell surface molecules such as E-cadherin and a number of ECM components [23]. These observations suggest that BMM modulates the secretion of MMP-2/7 but not MMP-1 to the basement membrane and promotes migration (Figure 3).

During re-epithelialization of the epidermis, ‘fibroplasia’ occurs in the dermis. Fbs increase proliferation around wound sites and form granulation tissue. Many types of growth factors or cytokines are secreted after injury, and these activate Fbs to differentiate into myofibroblasts, featured as contractile, differentiated, and capable of ECM production. In the last step of healing, the resolution phase, myofibroblasts are dramatically reduced by cell death, reducing ECM synthesis. However, it is not yet known whether myofibroblasts convert into the normal fibroblasts phenotype or a quiescent phenotype [5].

As a result of our experiments, we observed the activation to myofibroblasts by BMM, and it was induced by ROS generation via NOX4 activation. DPI inhibited Cyr61 expression at early time points; therefore, we speculated that the increase in ROS-related stress due to NOX4 stimulates Cyr61 expression and that this expression forms a positive feedback loop between Cyr61 and ROS. In a study by Kim et al. [24], Cyr61 (CCN1) functions to trigger cellular senescence by generating ROS through the induction of a RAC1-NOX1 complex in hepatic myofibroblasts [24]. Recently, CCN1 was identified as a pro-inflammatory factor that binds integrin α6β1 in keratinocytes [25]. Through this theory, we speculated that secreted Cyr61 from Fbs reaches keratinocytes and activates the integrin pathway, which is a specific receptor of FAK/Src. Further research is required to confirm these findings.

Angiogenesis is also an essential process for healing, as well as epithelialization and fibroplasia. Newly formed vessels offer micronutrients, amino acids, and oxygen to impaired skin [26]. To detect the vascularization effect of BMM, we investigated the vessels under the skin after burn-injury. Compared to the control group, BMM had no effect on angiogenesis.

Epithelial recovery, indicated by the upregulation of keratin-10, was found in the excisional wound model, as well as in an in vitro experiment. In the case of dermal recovery, the appearance of myofibroblasts labeled with FSP-1 was shown to be inconsistent in vitro and in vivo. The role of myofibroblasts during wound healing varies with each step. Myofibroblasts form granulation tissue at the proliferative stage, induce a contractile effect, and increase to synthesize ECM. Since excessive ECM deposition results in fibrosis and scarring, this phenomenon is reduced by apoptosis. These results suggest that BMM can help repair wounds by affecting the epidermis and dermis, and has no side effect such as scar formation. Rather, it inhibited scar formation (Figure 6 and Figure 7).

Interestingly, when HaCaT and Fbs are cultured together, HaCaT motility was significantly increased and BMM treatment further promoted. As shown in Figure 5C, this phenomenon was probably caused by synergistically increased TGF-β from HaCaT and Cyr61 from Fbs than from single cultured cells.

In recent research certifying the synergistic effect of a mixture of TMF and glycitin, we emphasized the interaction between HaCaTs and Fbs during wound repair [12]. Cytokines or growth factors secreted from HaCaTs and Fbs affect other cells and promote keratinocyte regeneration and fibroblast activation.

Here, we demonstrate the wound healing effect of BMM by stimulating re-epithelialization and dermal differentiation. As a mechanism for HaCaT migration by BMM, TGF-β-EMT and FAK/Src signaling were revealed and the Cyr61-NOX4 pathway was a core mechanism for the differentiation of Fbs. Furthermore, skin cells stimulated by BMM beneficially interact with one another and showed a synergistic effect. These findings suggest that the novel synthetic material, BMM, is a potential drug candidate for the treatment of skin injury.

## 4. Materials and Methods

### 4.1. Generation and Verification of BMM

Compound **I** (4-methoxybenzohydrazide) was purchased from Sigma-Aldrich (St. Louis, MO, USA) and used without purification. Compound **II** was prepared according to previously described methods [27]. Compound **I** (166 mg, 1 mmol) and a catalytic amount of concentrated hydrochloric acid were added to a solution of compound **II** (237 mg, 1 mmol) in ethanol. The reaction mixture was refluxed at 90 °C for 2 h. After completion of the reaction, the reaction mixture was cooled to room temperature to allow precipitation. The solid was filtered and washed with cold ethanol to yield a pure Compound **III** (yield: 50%, melting point: 238–240 °C). The synthesis procedure for Compound **III**, 1E,2E-1,2-bis((6-bromo-2H-chromen-3-yl)methylene)hydrazine, is shown in Figure 1A and its structure and numbering is shown in Figure 1B. The experimental methods for NMR spectroscopy and mass spectrometry to identify the compound followed a previously reported method [28]. Spectroscopic data are summarized as follows: 1H NMR (500 MHz, pyridine-d5) δ ppm: 8.34 (s, 2H, H-3a), 7.37 (dd, 2H, H-7, *J* = 2.4, 8.4 Hz), 7.35 (d, 2H, H-5, *J* = 2.4 Hz), 6.94 (s, 2H, H-4), 6.85 (d, 2H, H-8, *J* = 8.4), 5.31 (s, 4H, H-2); 13C NMR (125 MHz, pyridine-d5) δ ppm: 160.3 (C-3a), 155.1 (C-9), 133.8 (C-7), 131.6 (C-4), 130.9 (C-5), 130.6 (C-3), 124.8 (C-6), 118.3 (C-8), 113.8 (C-10), 65.0 (C-2); HRMS (*m*/*z*): calculated for [M + H]^+^: 472.9500.

BMM ([1E,2E-1,2-bis(6-bromo-2H-chromen-3-yl)methylene]hydrazine) was dissolved in dimethyl sulfoxide (DMSO) and stored at −20 °C (Stock = 20 μM).

### 4.2. Cell Culture

HaCaTs and primary human FBs were cultured in DMEM (Gibco, Carlsbad, CA, USA) supplemented with 10% FBS (Omega, Biel/Bienne, Switzerland) and 1% penicillin/streptomycin. Cells were incubated at 37 °C in 5% CO_2_.

### 4.3. MTT Assay

Cells (2 × 10^4^ cell/mL) were seeded in 96-well plates. The following day, cells were treated with DMSO (Sigma-Aldrich, St Louis, MO, USA) and BMM. After incubation for 24 or 48 h with HaCaT or Fbs, 10 μL of MTT solution (10 mg/mL) was added to each well, and cells were incubated for an additional 4 h at 37 °C. The medium was removed and replaced with 150 μL DMSO, and the plate was incubated for 30 min at room temperature with shaking. Absorbance was measured at 570 nm using a spectrophotometer. 

### 4.4. ECIS Proliferation Assay

ECIS is an experimental machine to analyze the proliferation or migration of cells in real time and show resistance values as a graph. The ECIS cell culture plate consists of six wells coated with metal on the bottom. As cells grow, resistance detected by metal is increased and is converted to impedance through a self-program. Two hundred microliters of electrode-stabilizing solution, composed of 10 mM l-cysteine, were added to each well and incubated at room temperature. After 10 min, the wells were washed using medium and the cells were seeded at a density of 2 × 10^4^ cell/mL. The following day, 0–20 μL of BMM was added to the wells and the cells were incubated for 48 h. Using the ECIS program, we transferred the Excel data sheet and calculated normalized impedance.

### 4.5. Cell Counting

The cell counting method used was one of the cell proliferation assays. A six-well plate was used for the cell counting assay (two wells per time point) and 5 × 10^3^ cells were seeded in each well. Cells were treated with 0–20 μL of BMM and incubated at 37 °C. After incubation for two, four, or six days, cells were collected using trypsin and centrifuged. Collected cells were suspended and counted using a hemocytometer. Every two days, medium containing the diluted sample was carefully changed.

### 4.6. Scratch Wound Healing Assay

Cells were seeded in 48-well plates at a density of 1 × 10^5^ cell/well for 24 h. A scratch was made on the cell monolayer by drawing a pipette tip across the well. The culture medium was supplemented with DMSO or BMM dose-dependently. At time 0 and 24 h post-treatment, wound closure was captured using an Olympus IX70 microscope (Tokyo, Japan) equipped with a digital camera at 40× magnification. The distance migrated was measured using the Image J software (Lviv, Ukraine), and calculated by comparing between the initial and final width of the scratch.

### 4.7. Matrigel Invasion Assay

We performed two types of Matrigel invasion assays: (1) single-culture or (2) co-culture. For single culture assays, 7 × 10^4^ cell/well of HaCaT or Fbs were seeded in the insert of a 12-well invasion assay kit (SPL), and the bottom chamber was filled with media. On the other hand, in case of the co-culture assays, HaCaT cells were seeded in the insert, and the Fbs were seeded into the bottom, using equal numbers of each. The membrane allows the exchange of media during incubation. After 24 h, media containing DMSO or BMM was added to the insert and bottom for 24 h. Cells on the upper side of the insert were removed using a cotton swab, and cells on the lower side of the insert were fixed with 4% formaldehyde. After washing with PBS, invaded cells were stained with a 1% crystal violet solution and photographs were acquired at 4× magnification using an Olympus IX70 microscope. Invasive ability was measured using the ImageJ program.

### 4.8. Western Blot

HaCaT, Fbs, or co-cultured cells were seeded at a density of 1 × 10^5^ each on 100 mm cell culture dishes and incubated for 24 h. These were treated with DMSO or BMM in a time or dose dependent manner. Total protein was extracted from treated cells using RIPA buffer, and the protein concentration was determined using the BCA Protein Assay Kit (Thermo Scientific, Walthm, MA, USA). To investigate the expression of secreted factors, conditioned medium was harvested and concentrated using an Amicon centrifugal filter (Merck Millipore, Burlington, MA, USA), and total protein concentration was measured using the Bradford assay. Equal amounts of protein (~30 μg/lane) were analyzed from each sample by resolving with 10% sodium dodecyl sulfate-polyacrylamide gel electrophoresis (SDS-PAGE). Protein loaded gels were transferred to NC membranes, and these were blocked in 5% skim milk with Tris-buffered saline Tween 20 (TBST) buffer, followed by incubation with primary antibodies at 4 °C overnight. Cytokeratin 10 (sc-53252), Cytokeratin 14 (sc-17104), MMP-7 (sc-8832), Cyr61 (sc-271217), Collagen-I (sc-25974),gp91-phox (sc-130543), FAK (sc-1688), p-FAK (sc-11765), C-Src (sc-130124), and GAPDH (sc-25778) antibodies were purchased from Santa Cruz Biotechnology (Santa Cruz, CA, USA); S100A4 (ab27957) antibody was purchased from ABcam (Bristol, UK); TGF-β (#3711), Slug (#9585), E-cadherin (#3195), Snail (#3879), p-Src (#2101), and MMP-2 (#4022) antibodies were purchased from Cell Signaling Technology (Danvers, MA, USA). NOX4 (NB110-58849) and MMP-1 (#444209) antibodies were purchased from Novus Biologicals (Littleton, CO, USA) and Calbiochem (San Diego, CA, USA), respectively. We used HRP-conjugated anti-mouse (K0211589, KOMABIOTECH, Seoul, Korea), anti-rabbit (K021178, KOMABIOTECH), and anti-goat (AP1079P, Millipore) as secondary antibodies. Proteins were detected using chemiluminescent reagent, ECL solution (W6002, Biosesang, Seongnam, Korea).

### 4.9. 2′,7′-Dichlorofluorescein Diacetate (DCF-DA) Assay

DCF-DA was used to detect intracellular ROS production. Dermal fibroblast cells (2 × 10^4^ cell/mL) were seeded in 96-well plates. To confirm the relationship between NOX and Cyr61, the cells were treated with BMM (20 μM) for 3 or 6 h or pre-treated with DPI (5 μM) for 1 h on the following day. Cells treated with H_2_O_2_ (100 μM) for 1 h before detection were used as a positive control, following which they were washed using 1× PBS. DCF-DA solution, which is a 1× PBS solution containing 1% FBS and 20 μM DCF-DA, was added to each well. Fluorescence was measured using a fluorescence plate reader (TECAN GENios, Grodig, Austria) with the following settings: excitation, 485 nm; emission, 535 nm. 

### 4.10. In Vivo Experiments

Six-week old male ICR mice (*n* = 6 for each group) were chosen for the experiment. All procedures were approved by the Animal Care and Use Committee at Jeju National University (permission number 2015-0033, 29 December 2015). First, the fur was removed with an electronic hair clipper and removal cream. Then, wounds were made on the middle of the back using a 6-mm biopsy punch. Wounds of mice in the experimental group were treated daily with 200 μL of BMM (200 μM) dissolved in butylene glycol for two weeks. Madecassol was used as a positive control and applied to wound sites in the same manner as BMM. Images were captured every two days to evaluate wound closure and the rate was calculated as a relative percentage of the original wound area, using the ImageJ program. At seven, nine, and 14 days post-wound, skin tissue was isolated and fixed using a 4% formaldehyde solution. Paraffin-embedded tissues were cut into 3-μm sections and stained using Hematoxylin-Eosin and Immunohistochemistry of Keratin 10 and FSP-1.

## Abbreviations

BMM	(1E,2E)-1,2-bis((6-bromo-2H-chromen-3-yl)methylene)hydrazine
Cyr61	Cysteine-rich angiogenic inducer 61
DCF-DA	2′,7′-dichlorfluorescein-diacetate
ECM	Extracellular matrix
EMT	Epithelial–mesenchymal transition
FAK	Focal adhesion kinase
MMP	Matrix metalloproteinase
NOX	Nicotinamide adenine dinucleotide phosphate oxidase
ROS	Reactive oxygen species
TGF-β	Transforming growth factor beta
